# Associations between common polymorphisms in *CYP2R1* and *GC*, Vitamin D intake and risk of colorectal cancer in a prospective case-cohort study in Danes

**DOI:** 10.1371/journal.pone.0228635

**Published:** 2020-02-03

**Authors:** Tine Iskov Kopp, Ulla Vogel, Vibeke Andersen

**Affiliations:** 1 Danish Cancer Society Research Center, Copenhagen, Denmark; 2 Department of Neurology, The Danish Multiple Sclerosis Registry, Rigshospitalet, Glostrup, Denmark; 3 National Research Centre for the Working Environment, Copenhagen, Denmark; 4 Focused Research Unit for Molecular Diagnostic and Clinical Research, IRS-Centre Sonderjylland, Hospital of Southern Jutland, Aabenraa, Denmark; 5 Institute of Regional Health Research-Center Sønderjylland, University of Southern Denmark, Odense, Denmark; 6 Institute of Molecular Medicine, University of Southern Denmark, Odense, Denmark; Brown University Warren Alpert Medical School, UNITED STATES

## Abstract

**Background:**

The association between vitamin D and incidence of colorectal cancer has been thoroughly investigated, but the results are conflicting. The objectives in this study were to investigate whether two functional polymorphisms in *GC* and *CYP2R1*, respectively, previously shown to predict vitamin D concentrations, were associated with risk of colorectal cancer; and further, to assess gene-environment interaction between the polymorphisms and intake of vitamin D through diet and supplementation in relation to risk of colorectal cancer.

**Methods:**

A nested case-cohort study of 920 colorectal cancer cases and 1743 randomly selected participants from the Danish prospective “Diet, Cancer and Health” study was performed. Genotypes *CYP2R1*/rs10741657 and *GC*/rs4588 were determined by PCR-based KASP^™^ genotyping assay. Vitamin D intake from supplements and diet was assessed from a validated food frequency questionnaire. Incidence rate ratios were estimated by the Cox proportional hazards model, and interactions between polymorphisms in *GC* and *CYP2R1* and vitamin D intake in relation to risk of colorectal cancer were assessed.

**Results:**

Neither of the two polymorphisms was associated with risk of colorectal cancer *per se*. Heterozygote carriage of *CYP2R1*/rs10741657 and *GC*/rs4588, and carriage of two risk alleles (estimated by a genetic risk score) were weakly associated with 9–12% decreased risk of colorectal cancer per 3 μg intake of vitamin D per day (IRR_*CYP2R1*/rs10741657_ = 0.88, 95% CI: 0.79–0.97; IRR_*GC*/rs4588_ = 0.91, 95% CI: 0.82–1.01, IRR_GRS2_ = 0.90, 95% CI: 0.81–0.99).

**Conclusions:**

The results suggest that genetic variation in vitamin D metabolising genes may influence the association between vitamin D intake, through food and supplementation, and risk of colorectal cancer.

**Clinical trial registry:**

NCT03370432. Registered 12 December 2017 (retrospectively registered).

## Introduction

The association between vitamin D and incidence of colorectal cancer has been thoroughly investigated with observational studies consistently demonstrating a significant inverse relationship between blood levels of the vitamin D metabolite 25-hydroxycholecalciferol (25(OH)D) and colorectal cancer incidence [1–3].

There is compelling evidence that inflammation is both an initiator and promotor of colorectal carcinogenesis [4,5]. The production of the active metabolite of vitamin D_3_, 1,25(OH)_2_D_3,_ also called calcitriol, begins in the skin, where 7-dehydrocholesterol is converted to cholecalciferol (vitamin D_3_) mediated by UVB radiation. Vitamin D_3_ requires further stepwise hydroxylations—first in the liver by CYP2R1, where it is converted to 25(OH)D—and then in the kidney to produce 1,25(OH)_2_D_3_. Calcitriol is known to exhibit many anti-inflammatory effects [6]. Therefore, vitamin D_3_ has been used as chemopreventive agent in healthy populations in three randomized, controlled trials (RCTs), although, without convincing success presumably due to short follow-up time, choice of study population, non-compliance and low sample size [7–9]. Therefore, other types of studies may be of use to shed light over this question.

Functional polymorphisms can be used as a tool for investigating molecular mechanisms in carcinogenesis when RCTs are inconclusive or not an option for ethical or economic reasons.

Inherited variants in *CYP2R1* (encoding CYP2R1) and *GC* (encoding the major vitamin D carrier protein in plasma, GC) have consistently been shown to modify 25(OH)D blood concentrations—the best biomarker of vitamin D concentration—both after UVB radiation and consumption of vitamin D_3_-fortified bread and milk in Danes [10]. Carriers of four risk alleles of *CYP2R1*/rs10741657 and *GC*/rs4588 polymorphisms had a mean concentration that was 20.9 nmol/L (~50%) lower than carriers of no risk alleles after UVB radiation in the Danish VitGen study [10]. Similarly, in the Danish VitmaD study [10–12], a baseline difference in 25(OH)D concentration was observed so that carriers of all four risk alleles had a significantly lower concentration of 25(OH)D in late summer compared to non-carriers. After 6 months consumption of vitamin D_3_-fortified bread and milk during winter, this difference was still present and, further, the largest percentage decrease in 25(OH)D concentration was seen for carriers of all four risk alleles with a decrease in 25(OH)D concentrations of ~20% whereas non-carriers actually had a ~5% increase in 25(OH)D concentrations.

Therefore, genetically determined differences in proteins and enzymes affecting transport and/or metabolism of vitamin D may affect circulating vitamin D level and hence, the risk of colorectal cancer. However, the amount of vitamin D that we ingest may interact with these genetic differences and therefore also affect the risk of colorectal cancer. Consequently, our aim was to investigate whether the two functional polymorphisms, shown to predict 25(OH)D level after vitamin D supplementation in the two Danish intervention studies [10–12], interact with vitamin D intake in relation to risk of colorectal cancer in a prospective, nested case-cohort study in Denmark. An interaction between the studied functional polymorphisms and vitamin D intake in relation to risk of colorectal cancer would lend to support the hypothesis that vitamin D is implicated in colorectal cancer carcinogenesis.

## Materials and methods

### Studied subjects

The “Diet, Cancer and Health” (DCH) Study is an ongoing Danish cohort study designed to investigate the relation between diet, lifestyle and cancer risk and has been thoroughly described elsewhere [13]. The cohort consists of 57,053 persons, recruited between December 1993 and May 1997. All the subjects were born in Denmark, and the individuals were 50 to 64 years of age and had no previous cancers at study entry. Blood samples, anthropometric measures and questionnaire data on diet and lifestyle were collected at study entry.

### Follow-up and endpoints

The present study used a nested case-cohort design. Follow-up was based on population-based cancer registries. Between 1994 and 31th December 2009, 1038 colorectal cancer cases were diagnosed. A sub-cohort of 1857 persons was randomly selected within the full cohort at time of entry into the cohort in agreement with the case-cohort study design [14] and, thus, without respect to time and disease status. Cases and sub-cohort were frequency-matched on gender. Due to the used design, with a priori sampling of the sub-cohort, 28 persons were both cases and sub-cohort, and these persons were kept in the analyses. 111 persons with missing genotype data and another 148 persons with missing information on relevant diet and lifestyle factors were excluded (including 1 person counting as both case and sub-cohort member) resulting in a total of 920 colorectal cancer cases and 1743 sub-cohort members for subsequent analyses with full information on both genotype and potential confounders (see S1 Fig). The present study group was previously described [15–21].

### Dietary and lifestyle questionnaire

Information on diet, lifestyle, weight, height, medical treatment, environmental exposures, and other socio-economic factors was collected at enrolment using questionnaires and interviews as described in details elsewhere [18,22–24]. In short, the food-frequency questionnaire (FFQ), assessed diet consumption in 12 categories of predefined responses, ranking from ‘never’ to ‘eight times or more per day’. The daily intake was then calculated by FoodCalc [25], using specifically developed standardized recipes and portion sizes. Information on vitamin D (and other dietary) supplements included open-ended questions on brand and doses, and categorical questions on frequency of intake, on the number of months of use during the last year and whether the supplement in question was used within the last month. Information on the contents of micronutrients in the different brands was obtained from producers or distributors of the specific products [13]. Part of the FFQ was validated by comparing mean calorie-adjusted intakes from the FFQ with two times seven day of weighed diet records resulting in correlation coefficients between 0.27 for vitamin A to 0.71 for calcium [22]. Vitamin D intake has not yet been validated. From the lifestyle questionnaire, data on smoking habits, hormone replacement therapy (HRT), history of alcohol intake and use of analgesics among others was obtained. Smoking status was classified as never, past or current. Persons smoking at least 1 cigarette daily during the last year were classified as smokers. Non-steroidal anti-inflammatory drug (NSAID) use (“Aspirin”, “Paracetamol”, “Ibuprofen”, or “Other pain relievers) was assessed as ≥ 2 pills per month during one year at baseline. Use of HRT among women was assessed as current, former or never user.

### Genotyping

Buffy coat preparations were stored at minus 150°C until use. DNA was extracted using a simple salting out procedure as described [26]. The DNA was genotyped by LGC (Middlesex, United Kingdom) by PCR-based KASP^™^ genotyping assay (www.lgcgroup.com). To confirm reproducibility, genotyping was repeated for 10% of the samples yielding 100% identity. During genotyping, DNA from cases and sub-cohort members was randomized and the case status of samples was blinded.

### Statistics

Deviation from Hardy-Weinberg equilibrium in the comparison group was assessed using a Chi-square test.

The data were sampled according to the case-cohort design and the weighted case-cohort approach was used for analyses [27]. Incidence rate ratios (IRR) for colorectal cancer were estimated by the Cox proportional hazards model. Age was used as the underlying time axis, which ensured that the estimation procedure was based on comparisons of individuals at the same age and the analyses were corrected for delayed entry, such that persons were considered under risk only from the age at enrolment in the cohort. Tests and 95% confidence intervals (CI) were based on Wald’s tests using the robust estimate of the variance-covariance matrix for the regression parameters in the Cox regression models [28].

All models were adjusted for baseline values of suspected risk factors for colorectal cancer selected *a priori*; that is, body mass index (BMI) (continuous, kg/m^2^), NSAID use (categorical, yes/no), HRT use (categorical, never/past/current (among women only)), smoking status (categorical, never/past/current), alcohol intake (continuous, g/day), intake of dietary fibre (continuous, g/day), and intake of red meat and processed meat (continuous, g/day). All analyses were stratified by gender, so that the baseline hazards were gender specific.

The linearity of all quantitative variables (exposure and covariate variables) was graphically evaluated in linear spline models with 3 or 9 boundaries placed at the quartiles or deciles among cases; and since no sign of deflection was observed, all variables were entered into the Cox regression models [29,30].

Alleles associated with high 25(OH)D concentrations [10] was used as references in the analyses. Variant genotypes were combined in the interaction analysis to maximize the statistical power since no recessive effect was observed. Additionally, to compare the results to the VitGen study where carriers of AG or AA of *CYP2R1*/rs10741657 had the highest increase in 25(OH)D concentration after UVB exposure (28.8 and 30.7 nmol/L, respectively, compared to only 21.7 nmol/L for GG carriers, p = 0.024), we also present risk estimates for AA+AG carriers versus GG carriers for *CYP2R1*/rs10741657. A genetic risk score (GRS) was calculated as the sum of the number of risk alleles (range: 0–4), that is, the sum of the number of G alleles of *CYP2R1*/rs10741657 and A alleles of *GC*/rs4588 based on the study by Nissen *et al*. [10].

Multiplicative interaction between vitamin D intake and SNPs or GRS was tested using product terms of the continuous vitamin D intake variable (3 μg/day) and genotype in the weighted Cox regression models adjusted for the above-mentioned covariates.

A p-value <0.05 was considered statistically significant. All analyses were performed using the statistics program SAS, version 9.4 (SAS Institute, Cary, NC).

### Ethics

All participants gave verbal and written informed consent. The DCH study was approved by the National Committee on Health Research Ethics (journal nr. (KF) 01-345/93) and the Danish Data Protection Agency.

## Results

Baseline characteristics of colorectal cancer cases and sub-cohort members including colorectal cancer risk factors are presented in Table 1 as partly published previously [15–18].

**Table 1:** Baseline characteristics of the study participants by selected demographic and established colorectal cancer risk factors.

Variable	Cases		Sub-cohort members		IRR^1^ (95% CI)
	N (%)	Median (5–95%)	N (%)	Median (5–95%)	
Total	920 (35)		1743 (65)		
Sex					
Men	516 (56)		935 (54)		
Women	404 (44)		808 (46)		
Age at inclusion (years)		58 (50–64)		56 (50–64)	
BMI (kg/m^2^)		26.3 (20.6–34.3)		25.6 (20.5–35.9)	1.05 (1.01–1.10)^5^
Food intake (g/day)					
Alcohol^2^		15.1 (1.2–71.4)		14.2 (1.2–65.7)	1.04 (1.00–1.08)^6^
Dietary fiber		19.8 (10.5–32.7)		20.6 (10.7–34.2)	0.88 (0.78–1.01)^7^
Red and processed meat		112.3 (47.1–233.4)		109.4 (42.5–236.3)	1.03 (0.99–1.07)^8^
Vitamin D from diet (μg/day)		4.4 (1.9–8.8)		4.5 (1.9–9.0)	1.01 (0.87–1.18)^9^
Vitamin D from supplements (μg/day)^3^		4.2 (0.5–11.4)		4.2 (0.4–10.0)	-
Total vitamin D (μg/day)		5.8 (2.3–12.7)		6.0 (2.2–14.0)	0.94 (0.86–1.02)^10^
Smoking status					
Never	277 (30)		574 (33)		1.00 (ref.)
Past	280 (30)		519 (30)		1.05 (0.84–1.30)
Current	363 (40)		650 (37)		1.13 (0.92–1.39)
NSAID use^4^					
No	641 (70)		1204 (69)		1.00 (ref.)
Yes	279 (30)		539 (31)		1.00 (0.83–1.20)
HRT use among women					
Never	244 (60)		423 (52)		1.00 (ref.)
Past	52 (13)		127 (16)		0.64 (0.44–0.93)
Current	108 (27)		258 (32)		0.71 (0.53–0.95)

values are expressed as medians (5th and 95th percentiles) or as fractions (%). IRR, incidence rate ratio; CI, confidence interval; BMI, body mass index; NSAID, non-steroidal anti-inflammatory drug; HRT, hormone replacement therapy

^1^ IRRs for colorectal cancer estimated by the Cox proportional hazards model mutually adjusted for all variables, with age as the underlying time axis, and stratified by gender, so that the baseline hazards are gender specific. 95% CI are based on Wald’s tests.

^2^ Among current drinkers (N_cases_ = 897; N_sub-cohort_ = 1702).

^3^ Among supplement users (N_cases_ = 392; N_sub-cohort_ = 817).

^4^ NSAID use is defined as ≥2 pills per month for one year.

^5^ Risk estimate per 2 kg/m^2^ increment of BMI.

^6^ Risk estimate for the increment of 10 g alcohol per day.

^7^ Risk estimate for the increment of 10 g dietary fibres per day.

^8^ Risk estimate for the increment of 25 g red and processed meat per day.

^9^ Risk estimate for the increment of 3 μg vitamin D from the diet per day.

^10^ Risk estimate for the increment of 3 μg vitamin D from diet and supplements per day.

Overall, vitamin D intake (from supplements and diet) was associated with a statistically non-significant 6% reduced risk of colorectal cancer per 3 μg per day (IRR = 0.94, 95% CI: 0.86–1.02).

Among sub-cohort members, the genotype distributions of the studied polymorphisms were in Hardy–Weinberg equilibrium. Neither of the two polymorphisms was associated with risk of colorectal cancer *per se* (Table 2).

**Table 2:** Risk of colorectal cancer in relation to the studied polymorphisms.

	N_cases_/N_sub-cohort_	IRR (95% CI)^1^	IRR (95% CI)^2^	p-value^3^
*CYP2R1*/rs10741657				
AA	183/313	1.00 (ref.)	1.00 (ref.)	
AG	439/845	0.90 (0.72–1.12)	0.91 (0.72–1.13)	0.38
GG	298/585	0.89 (0.70–1.12)	0.90 (0.71–1.15)	0.40
AG+GG	737/1430	0.90 (0.73–1.10)	0.90 (0.73–1.12)	0.35
AA+AG vs. GG	622/1158	1.04 (0.88–1.24)	1.03 (0.87–1.23)	0.73
*GC*/rs4588				
CC	491/914	1.00 (ref.)	1.00 (ref.)	
CA	350/708	0.92 (0.77–1.09)	0.93 (0.78–1.10)	0.38
AA	79/121	1.12 (0.82–1.54)	1.13 (0.82–1.55)	0.46
CA+AA	429/829	0.95 (0.81–1.12)	0.96 (0.81–1.13)	0.60

BMI, body mass index; CI, confidence interval; HRT, hormone replacement therapy; IRR, incidence rate ratio; NSAID, non-steroidal anti-inflammatory drug.

^1^ IRRs for colorectal cancer estimated by the Cox proportional hazards model with age as the underlying time axis, and stratified by gender, so that the baseline hazards are gender specific. 95% CI are based on Wald’s tests.

^2^ In addition, adjusted for smoking status, alcohol intake, HRT status (women only), BMI, use of NSAID, intake of red and processed meat, and dietary fibre.

^3^ *p*-value for the adjusted risk estimates.

The combined effect of *CYP2R1*/rs10741657 and *GC*/rs4588 was investigated using a GRS calculated as the sum of the number of G alleles of *CYP2R1*/rs10741657 and A alleles of *GC*/rs4588. However, we found no association between the GRS and risk of colorectal cancer *per se* (Table 3).

**Table 3:** Risk of colorectal cancer in relation to GRS.

GRS	N_cases_/N_sub-cohort_	IRR (95% CI)^1^	IRR (95% CI)^2^	*p*-value^3^
0	102/154	1.00 (ref.)	1.00 (ref.)	
1	290/582	0.74 (0.55–0.99)	0.73 (0.54–0.99)	
2	353/667	0.81 (0.60–1.08)	0.81 (0.61–1.09)	
3+4	175/340	0.75 (0.54–1.03)	0.76 (0.55–1.04)	0.20

The GRS (range: 0–4) was calculated as the sum of the number of G alleles of *CYP2R1*/rs10741657 and A alleles of *GC*/rs4588. BMI, body mass index; CI, confidence interval; Genetic Risk Score, GRS; HRT, hormone replacement therapy; IRR, incidence rate ratio; NSAID, non-steroidal anti-inflammatory drug.

^1^ IRRs for colorectal cancer estimated by the Cox proportional hazards model with age as the underlying time axis, and stratified by gender, so that the baseline hazards are gender specific. 95% CI are based on Wald’s tests.

^2^ In addition, adjusted for smoking status, alcohol intake, HRT status (women only), BMI, use of NSAID, intake of red and processed meat, and dietary fibre.

^3^ *p*-value for trend for the adjusted risk estimates.

Interaction between vitamin D intake and genotype was assessed in relation to risk of colorectal cancer in Table 4.

**Table 4:** Risk of colorectal cancer for vitamin D intake (3 μg/day) in relation to the studied polymorphisms.

	N_cases_/N_sub-cohort_	IRR (95% CI)^1^	IRR (95% CI)^2^	*p*-value^3^
*CYP2R1*/rs10741657				
AA	183/313	0.92 (0.80–1.06)	0.92 (0.80–1.07)	
AG	439/845	0.86 (0.78–0.95)	0.88 (0.79–0.97)	
GG	298/585	1.05 (0.93–1.20)	1.07 (0.94–1.22)	0.04
AG+GG	737/1430	0.93 (0.86–1.00)	0.95 (0.88–1.03)	0.75
AA+AG vs. GG	622/1158	0.88 (0.81–0.95)	0.89 (0.82–0.97)	0.007
*GC*/rs4588				
CC	491/914	0.97 (0.88–1.07)	0.99 (0.89–1.09)	
CA	350/708	0.89 (0.80–0.99)	0.91 (0.82–1.01)	
AA	79/121	0.87 (0.71–1.08)	0.89 (0.72–1.10)	0.44
CA+AA	429/829	0.89 (0.81–0.97)	0.90 (0.82–0.99)	0.20

BMI, body mass index; CI, confidence interval; HRT, hormone replacement therapy; IRR, incidence rate ratio; NSAID, non-steroidal anti-inflammatory drug.

^1^ IRRs for colorectal cancer estimated by the Cox proportional hazards model with age as the underlying time axis, and stratified by gender, so that the baseline hazards are gender specific. 95% CI are based on Wald’s tests.

^2^ In addition, adjusted for smoking status, alcohol intake, HRT status (women only), BMI, use of NSAID, intake of red and processed meat, and dietary fibre.

^3^ *p*-value for interaction between genotype and 3 μg Vitamin D per day for the adjusted risk estimates.

For *GC*/rs4588, heterozygote carriage was associated with 9% decreased risk of colorectal cancer per 3 μg vitamin D ingested per day with borderline statistical significance (IRR = 0.91, 95% CI: 0.72–1.01, p = 0.07). Homozygous carriers were at 11% reduced risk of colorectal cancer, but the estimate did not reach statistical significance due to the smaller number of cases and controls in this group. Heterozygote carriage of *CYP2R1*/rs10741657 was associated with a 12% decrease in colorectal cancer risk per 3 μg vitamin D ingested per day (IRR = 0.88, 95% CI: 0.79–0.97). The slopes of the curves describing the relationship between vitamin D intake and colorectal cancer risk for carriers of the different genotypes, were only significantly different from each other for *CYP2R1*/rs10741657 (p for interaction (p_int_) = 0.04). Again, homozygous AA-carriers had a similar 8% reduced risk of colorectal cancer by vitamin D intake that was not statistically significant probably due to the smaller numbers of cases and controls in this group. To compare the results to the VitGen study, we therefore also calculated risk estimates for AA+AG carriers versus GG carriers (Table 4). Being AA or AG carriers was associated with a 11% decreased risk of colorectal cancer per 3 μg vitamin D ingested per day (IRR = 0.89, 95% CI: 0.82–0.97) whereas being GG carrier was not associated with risk of colorectal cancer for vitamin D intake (IRR = 1.07, 95% CI: 0.94–1.22). The slopes between the two curves (AA+AG vs GG) differed significantly (p_int_ = 0.007). In a supplementary analysis (S1 Table), gene-gene-diet interaction was analysed and showed that variant carriage of both polymorphisms was associated with a 18% decreased risk of colorectal cancer per 3 μg vitamin D ingested per day (IRR = 0.82, 95% CI: 0.73–0.93) whereas other combinations of the polymorphisms were not associated with risk of colorectal cancer for vitamin D intake (p_int_ = 0.04 on a multiplicative scale)

No overall interaction between GRS and vitamin D intake in relation to risk of colorectal cancer was found (Table 5); but again, carriers of 2 risk alleles were at 10% decreased risk of colorectal cancer per 3 μg vitamin D ingested per day (IRR = 0.90, 95% CI: 0.81–0.99).

**Table 5:** Interaction between vitamin D intake (3 μg/day) and GRS in relation to colorectal cancer risk.

GRS	N_cases_/N_sub-cohort_	IRR (95% CI)^1^	IRR (95% CI)^2^	*p*-value^3^
0	102/154	1.05 (0.84–1.31)	1.03 (0.82–1.30)	
1	290/582	0.90 (0.80–1.02)	0.92 (0.81–1.05)	
2	353/667	0.88 (0.80–0.97)	0.90 (0.81–0.99)	
3+4	175/340	1.05 (0.88–1.25)	1.07 (0.90–1.28)	0.27

The GRS (range: 0–4) was calculated as the sum of the number of G alleles of *CYP2R1*/rs10741657 and A alleles of *GC*/rs4588. BMI, body mass index; CI, confidence interval; Genetic Risk Score, GRS; HRT, hormone replacement therapy; IRR, incidence rate ratio; NSAID, non-steroidal anti-inflammatory drug.

^1^ IRRs for colorectal cancer estimated by the Cox proportional hazards model with age as the underlying time axis, and stratified by gender, so that the baseline hazards are gender specific. 95% CI are based on Wald’s tests.

^2^ In addition, adjusted for smoking status, alcohol intake, HRT status (women only), BMI, use of NSAID, intake of red and processed meat, and dietary fibre.

^3^ *p*-value for interaction between GRS and 3 μg Vitamin D per day for the adjusted risk estimates.

## Discussion

In the present study, we found a non-significant inverse association between colorectal cancer and vitamin D intake in accordance with several other studies investigating associations between the vitamin D biomarker 25(OH)D, UVB exposure, dietary or supplement intake and risk of colorectal cancer (reviewed in [31]). While we were unable to find an association between functional polymorphisms predicting genetically determined low vitamin D levels and increased colorectal cancer risk, we found, however, significant interaction between the functional polymorphism *CYP2R1*/rs10741657 and vitamin D intake in relation to risk of colorectal cancer. We also found weak association between heterozygous carriage of *GC*/rs4588 and of two risk alleles, and vitamin D intake in relation to risk of colorectal cancer.

Vitamin D is supplemented as cholecalciferol. *CYP2R1*/rs10741657 is a promotor polymorphism, leading to lowered synthesis of CYP2R1 for the variant G-allele [32], presumably resulting in lowered conversion rate of cholecalciferol into 25(OH)D. *CYP2R1*/rs10741657 has consistently been associated with 25(OH)D concentrations in several populations [11,12,32–36] such that carriers of the G-allele have the lowest 25(OH)D serum concentrations. Thus, the found interaction suggests that vitamin D intake is protective against colorectal cancer in individuals with genetically determined high CYP2R1 enzyme levels, but not among individuals with genetically determined low CYP2R1 enzyme levels. The interaction lends some support to the notion that vitamin D is implicated in colorectal cancer carcinogenesis. Correspondingly, another recent study found no association between a functional polymorphism in *GC* and colorectal cancer risk, but found evidence of effect modification of dietary vitamin D intake on the association between genotype and colorectal cancer risk [37].

The polymorphisms investigated in the present study have been thoroughly examined in both experimental and observational studies with regards to their effect on 25(OH)D concentrations. They may therefore be considered as being strong predictors of vitamin D level. The *GC*/rs4588 polymorphism causes a missense substitution of Thr to Lys resulting in a lower affinity to both 25(OH)D and 1,25(OH)_2_D [38]. Several studies have reported lower 25(OH)D concentrations among carriers of the variant A allele of *GC*/rs4588 compared to CC homozygotes [11,12,39–46]. Since GC is the major vitamin D carrier protein in plasma, the lower 25(OH)D concentrations among carriers of the variant A allele are likely caused by the lower binding affinity of the variant GC protein [47].

In the VitmaD study, baseline measurements were conducted in a Danish population at study entry during late summer, and the intervention with vitamin D supplementation in bread and milk took place during winter. The VitGen study was also conducted during winter in a Danish population. The studies showed that a GRS consisting of the functional polymorphisms in *CYP2R1* and *GC* predicted 25(OH)D concentrations both at baseline and after supplementation by both UVB exposure and by enrichment of food in a Danish population consisting of both adults and children [10]. This emphasizes that the studied functional polymorphisms of the GRS are very consistently associated to 25(OH)D concentrations after both dietary supplementation and UVB exposure. The current prospective study is also conducted in a Danish population. If we consider the SNPs as functional, the participants carrying the variant alleles are exposed to low vitamin D levels their entire life. We would therefore expect to find an association between the GRS and colorectal cancer if vitamin D protects strongly against colorectal cancer. However, the studied polymorphisms predicting genetically determined low 25(OH)D concentrations were not associated with increased risk of colorectal cancer *per se*. On the other hand, we found interaction between *CYP2R1*/rs10741657 and vitamin D intake in relation to risk of colorectal cancer. In addition, heterozygote carriers of *GC*/rs4588 were at statistically significantly reduced risk of colorectal cancer by vitamin D intake. Although homozygous carriers of the A-allele numerically had the same risk reduction, it was not statistically significant probably due to the lower numbers in this genotype group. These results place vitamin D in colorectal cancer carcinogenesis. The results are consistent with the VitGen study where AA and AG carriers of *CYP2R1*/rs10741657 and heterozygote carriers of *GC*/rs4588 had the highest increase in 25(OH)D after UVB exposure compared to GG carriers for *CYP2R1*/rs10741657 and CC and AA carriers for *GC*/rs4588 [10]. Likewise, in the VitmaD study, AA and AG carriers of *CYP2R1*/rs10741657 and CC carriers of *GC*/rs4588 had the highest 25(OH)D concentration at baseline in late summer and after 6 months consumption of vitamin D_3_-fortified bread and milk during wintertime. If CC and CA genotypes of the *GC*/rs4588 polymorphisms are combined in the present study, a further risk reduction was seen. We therefore consider our findings in this study to agree with both the VitmaD and VitGen studies [10].

Our results could indicate that vitamin D intake may protect against colorectal cancer among persons with genetically determined intermediate efficient vitamin D conversion and distribution (GRS 1–2). Indeed, persons with genetically determined highly efficient vitamin D conversion and distribution (GRS 0)—associated with high vitamin D serum level—do not benefit from vitamin D supplementation. Also, carriers of all four risk alleles (poorly efficient) have no benefit from vitamin D supplementation. In the controlled vitamin D supplementation study (VitmaD), carriers of all four risk alleles were the group with the lowest 25(OH)D concentrations and the group who benefitted the least from supplementation (VitmaD) and UVB exposure (VitGen) [10]. The results from this study could thus suggest that carriers of three and four risk alleles require much higher vitamin D intakes to achieve reduced risk of colorectal cancer simply because their vitamin D uptake is inefficient as documented in the VitmaD and the VitGen studies. The lack of effect of vitamin D intake in the group with GRS 3+4 in relation to risk of colorectal cancer in the present study may, therefore, be consistent with the previous two studies.

This hypothesis is partly in agreement with the study by Zhu and colleagues [37], who investigated another functional *GC* polymorphism and the association with colorectal cancer and possible effect modification by vitamin D intake. They found that carriage of a “high serum 25(OH)D allele” benefitted the most from a high vitamin D intake, whereas those with a “low serum 25(OH)D allele” had little benefit from high vitamin D intake, which they hypothesised could be due to their low affinity and abundance of GC that might influence the function of vitamin D.

Consequently, it is possible that not all persons benefit from vitamin D supplementation and that this is determined by a persons’ vitamin D metabolizing phenotype, that is, the ability to bind to GC protein, 25(OH)D and/or 1.25(OH)_2_D. Additional studies should be addressed to investigate the role of individual vitamin D metabolizing phenotypes on the health-related effect of vitamin D supplementation.

There are several limitations to this study. First of all, the effect sizes in this study are small which require larger sample sizes to detect weak gene-environment interactions [48]. On the other hand, the studied polymorphisms have a high minor allele frequency (0.28–0.44) and we have used dominant models, which require much smaller sample sizes to detect even weak interactions [48]. Also, the results are consistent with the two controlled studies conducted in a population similar to the DCH cohort. Secondly, the intake of vitamin D is very low in the DCH cohort as in the Danish population in general [49] and this low intake may not be sufficient to detect an effect of the polymorphisms. Thirdly, dietary intake based on FFQ is affected by measurement errors introduced by either the participants or the FFQ which may have affected our results, but this potential misclassification is probably nondifferential and therefore has biased results towards unity. Lastly, we did not consider other possible sources of vitamin D (e.g. sun light and outdoor activity) in the analyses which could explain non-significant results.

Another explanation for the protective effect of vitamin D intake found in many previous studies could simply be that vitamin D intake is a proxy of a heathy life style in general and that the weak effects that we see in the present study therefore are caused by other non-vitamin D related mechanisms. This is plausible since a healthy diet and lifestyle is known to protect against colorectal cancer [50–52]. However, the observed gene-environment interaction between *CYP2R1*/rs10741657 and vitamin D intake argues against this.

The advantage of this study is the prospective study design which is well suited for gene-environment interaction analyses due to the collection of dietary and life style factors before diagnosis, eliminating the risk of recall bias. Changes in dietary and life style habits during follow-up are, however, possible, but are not expected to result in differential misclassification between cases and the comparison group. The DCH cohort is very homogenous eliminating population specific genetics and dietary patterns seen in larger multicentre studies. A disadvantage of the prospective study is the limited power to detect gene-environment interactions.

In conclusion, genetic variation in vitamin D metabolising genes may influence the association between vitamin D intake through food and supplementation and risk of colorectal cancer. We hypothesise that persons with genetically determined intermediate efficient vitamin D conversion and distribution may benefit the most from vitamin D intake. Replication of our findings in larger cohorts is warranted and could potentially leads to individual recommendation of vitamin D dosage.

## Supporting information

S1 Fig Flow chart of study participants. (DOCX)

S1 Table Interaction between combination of *CYP2R*/rs10741657 and *GC*/rs4588 and intake of 3 μg vitamin D per day in relation to risk of colorectal cancer. (DOCX)

## List of abbreviations

25(OH)D: 25-hydroxycholecalciferol
CI: Confidence intervals
DCH: Diet, Cancer and Health”
FFQ: Food-frequency questionnaire
HRT: Hormone replacement therapy
IRR: Incidence rate ratios
NSAID: Non-steroidal anti-inflammatory drug
RCT: Randomized clinical trial
SNP: Single nucleotide polymorphism
