# Supplementary material for: Associations between common polymorphisms in CYP2R1 and GC, Vitamin D intake and risk of colorectal cancer in a prospective case-cohort study in Danes
Source: PLoS One. 2020 Feb 3;15(2):e0228635. doi: 10.1371/journal.pone.0228635 (PMC6996822; doi:10.1371/journal.pone.0228635)
Supplement: S1 Fig — (DOCX) [file pone.0228635.s001.docx]

114 sub-cohort members were excluded due to missing information on genotype and/or potential confounders.

A total of 1743 sub-cohort members were eligible for analyses.

118 colorectal cancer cases were excluded due to missing information on genotype and/or potential confounders.

28 participants were both cases and controls.

A total of 920 colorectal cancer cases were eligible for analyses.

Between 1994 and 31th December 2009, 1038 colorectal cancer cases were diagnosed.

A sub-cohort of 1857 participants was randomly selected within the full cohort at time of entry into the cohort as controls.

27,179 (34%) men and 29,875 (37%) women accepted the invitation and were recruited.

Between December 1993 and May 1997, 160,725 persons aged 50–64 years, born in Denmark, living in the Copenhagen or Aarhus areas and having no previous cancers at the time of invitation, were invited to participate in the study.
