# Supplementary material for: Associations between common polymorphisms in CYP2R1 and GC, Vitamin D intake and risk of colorectal cancer in a prospective case-cohort study in Danes
Source: PLoS One. 2020 Feb 3;15(2):e0228635. doi: 10.1371/journal.pone.0228635 (PMC6996822; doi:10.1371/journal.pone.0228635)
Supplement: S1 Table — (DOCX) [file pone.0228635.s002.docx]

**Table S1. Interaction between combination of *CYP2R*/rs10741657 and *GC*/rs4588 and intake of 3 µg Vitamin D per day in relation to risk of colorectal cancer.**

|  | ***GC*/rs4588** | | ***GC*/rs4588** | | ***GC*/rs4588** | | **p-value^3^** |
| --- | --- | --- | --- | --- | --- | --- | --- |
|  | **CC**  **N_cases_/N_sub-cohort_** | **CA+AA**  **N_cases_/N_sub-cohort_** | **CC**  **IRR (95% CI)^1^** | **CA+AA**  **IRR (95% CI) ^1^** | **CC**  **IRR (95% CI)^2^** | **CA+AA**  **IRR (95% CI) ^2^** |  |
| *CYP2R*/rs10741657  GG  AG+AA | 167/306  324/608 | 131/279  298/550 | 1.04 (0.88-1.23)  0.94 (0.83-1.06) | 1.07 (0.88-1.30)  0.82 (0.74-0.92) | 1.04 (0.88-1.24)  0.93 (0.82-1.05) | 1.08 (0.89-1.31)  0.82 (0.73-0.93) | 0.04 |

Note: BMI, body mass index; CI, confidence interval; HRT, hormone replacement therapy; IRR, incidence rate ratio; NSAID, non-steroidal anti-inflammatory drug.

^1^ IRRs for CRC estimated by the Cox proportional hazards model with age as the underlying time axis, and stratified by gender, so that the underlying hazards are gender specific. 95% CI are based on Wald’s tests.

^2^ In addition, adjusted for smoking status, alcohol intake, HRT status (women only), BMI, use of NSAID, intake of red and processed meat, and dietary fibre.

^3^ *p*-value for interaction on a multiplicative scale.
